# Poison-Centre Scorpion-Sting Records in Turkey, 2014–2020: A Data-Quality Audit

**DOI:** 10.3390/toxins18100454

**Published:** 2026-10-19

**Authors:** Yıldırım Cesaretli, Özcan Özkan

**Affiliations:** 1 Department of Medical Pharmacology, Gülhane Faculty of Medicine, University of Health Sciences, Ankara 06018, Turkey; 2 Department of Biology, Faculty of Science, Çankırı Karatekin University, Çankırı 18100, Turkey; ozcanozkan@karatekin.edu.tr

**Keywords:** scorpion sting, poison control centre, routinely collected health data, toxicovigilance, surveillance, Turkey

## Abstract

Poison-centre records can inform toxicovigilance, but their interpretation depends on stable definitions and complete fields. We audited scorpion-sting data published by Turkey’s National Poison Control Centre (UZEM) and mapped record-design features in international publications. The detailed 2014–2018 appendix was analysed separately from 2019–2020 tables. It contained 7055 records; 51.0% involved males, 48.8% females, and 0.2% had unreported sex. May–September accounted for 74.1%, with July highest (17.9%). The source’s unequal age intervals initially favoured wider adult categories; after harmonisation into 10-year bands, ages 0–9 became the largest band in every year and represented 21.8% overall. Aggregate tables separately reported 1451 records in 2019 and 1729 in 2020. The audit covered 191 analytic ledger entries across 39 variables and identified numerical conflicts, changing definitions, and gaps in clinical linkage. Clinical tables were available only for 2017–2018, with no symptom recorded for 50.6% and 37.6% of annual records, respectively. Management fields documented recommendations and limited post-antivenom feedback, but not complete linked administration, severity, disposition, or outcomes. We propose a draft field framework prioritising event identity, clinical trajectory, treatment, disposition, and outcome. The findings describe poison-centre reporting activity rather than population risk.

## 1. Introduction

Scorpion stings are an important environmental toxicology problem in tropical and subtropical regions. Most stings cause local pain and inflammation, but systemic autonomic, cardiovascular, respiratory, or neurological toxicity may occur, particularly in children and after exposure to medically important species. The observed burden varies with scorpion fauna, climate, housing and occupational conditions, access to care, and the organisation of reporting systems [1,2].

**Figure 1:**
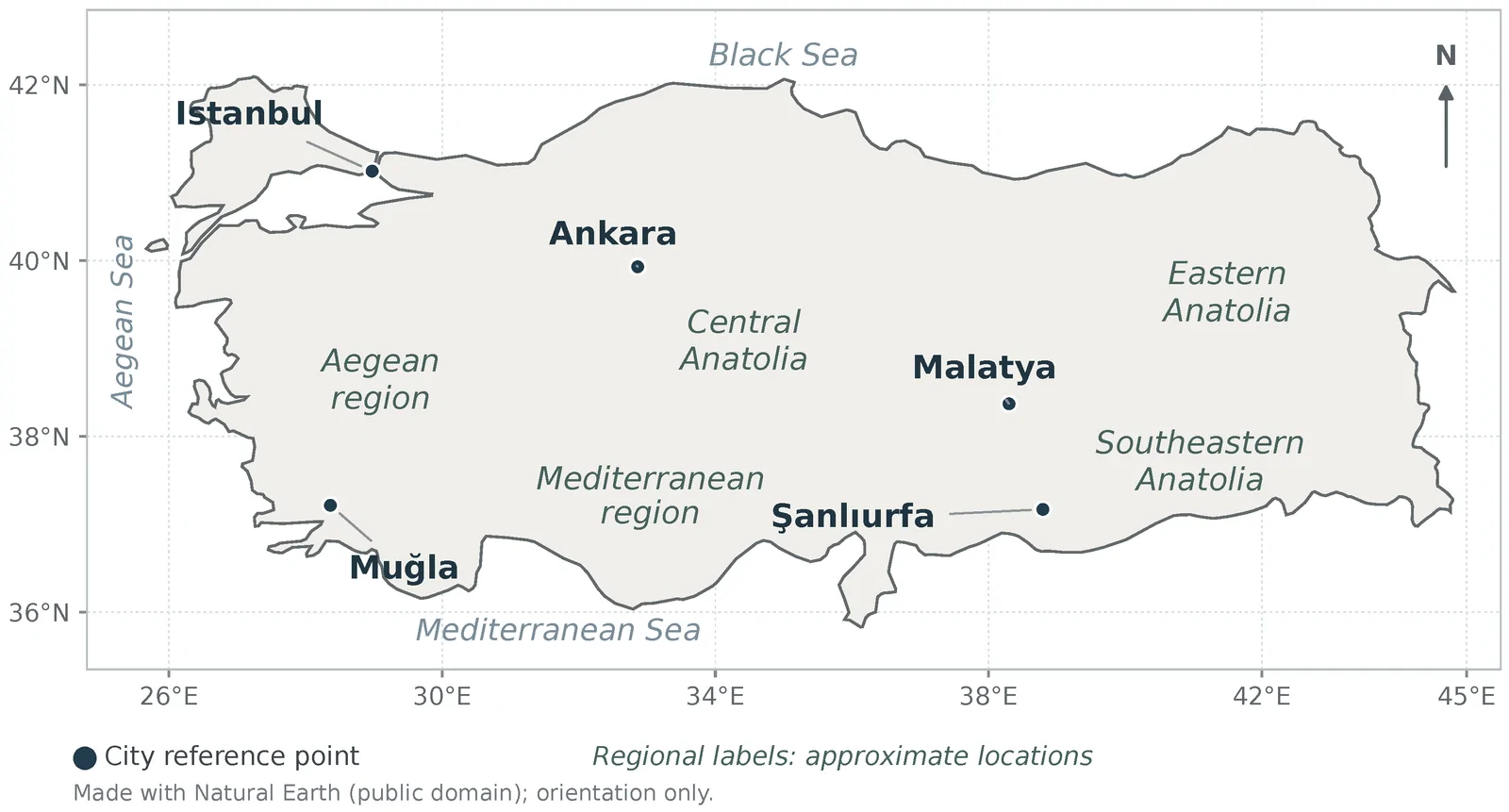
Geographical orientation to the Turkish locations and broad regions mentioned in the text. Points locate city centres; manuscript province counts refer to reporting-associated provinces, not city populations. Regional labels indicate approximate positions and do not delineate administrative boundaries, incidence, or species distributions. Base map and city coordinates: Natural Earth (public domain), plotted with Python 3.12.14/Matplotlib 3.11.2 using AI-assisted code.

Turkey has a taxonomically diverse scorpion fauna whose taxonomy continues to be revised, represented by Buthidae, Euscorpiidae, Iuridae, and Scorpionidae. The places and broad regions discussed below are located in Figure 1. Representative medically relevant buthids documented in the country include *Androctonus turkiyensis* in Şanlıurfa, *Leiurus abdullahbayrami* in southeastern Turkey, *Aegaeobuthus gibbosus* in southwestern and western Anatolia, and members of the *Mesobuthus* complex in eastern and southeastern regions [3,4,5,6]. Taxonomic revision is clinically relevant: Şanlıurfa populations reported as *A. crassicauda* in older Turkish studies were subsequently separated as *A. turkiyensis*, while Turkish records of *L. quinquestriatus* were reassigned to *L. abdullahbayrami* [4,5]. Older clinical names are retained below only when reporting the terminology used by the original studies.

Species-specific clinical evidence remains limited because the offending scorpion was seldom collected or identified. National poison-centre data described local pain, hyperaemia, oedema, and numbness, together with systemic hyperthermia, nausea or vomiting, tachycardia, shivering, and lethargy, but did not link these findings to confirmed species [7]. In a southeastern paediatric series, specimens then identified as *A. crassicauda* accounted for 24 of 52 stings; tachycardia and cold extremities were frequent, and 20 children required intensive care for serious envenomation [8]. Regional paediatric reports additionally recorded burning, sweating, dry mouth, thirst, and blood-pressure disturbances [9]. Severe *L. abdullahbayrami* envenomation has been species-confirmed in a child with confusion, hallucinations, hypotension, tachycardia, cardiomyopathy, and acute pulmonary oedema [10]. A recent paediatric series also reported vomiting, sweating, sinus tachycardia, and supraventricular tachycardia among systemic manifestations [11]. These findings establish a spectrum from local toxicity to life-threatening autonomic, cardiac, respiratory, and neurological involvement, while also showing why contemporary species verification is needed.

Turkey has several complementary but non-equivalent sources of scorpion-sting data. National Poison Control Centre records for 1995–2004 included 930 records; the original publication reported a July peak and identified ages 20–29 as the largest published age category [7]. A separate Ministry of Health notification study recorded 24,261 stings in 2005, with most notifications originating from Southeastern Anatolia, the Mediterranean region, and the Aegean region [12]. More recently, a study based on provincial health directorate data reported 30,769 stings from 38 provinces during 2015–2020 [13]. These markedly different counts reflect different populations, reporting routes, inclusion criteria, and degrees of coverage; they cannot be ordered as a simple national time trend.

The Turkish Ministry of Health published a consolidated UZEM report covering 2014–2020 [14]. Within that publication, however, the dedicated scorpion-sting appendix provides detailed demographic, temporal, geographical, exposure, clinical, and treatment information only for 2014–2018. The 2019 and 2020 annual reports contain scorpion counts within broader poison-group tables but do not provide comparable scorpion-specific cross-tabulations. The source therefore offers an opportunity not only to describe scorpion-sting reporting patterns but also to examine the limitations of using routinely collected poison-centre data as national surveillance data.

The consolidated UZEM publication was the sole primary data source analysed in this study. Its detailed 2014–2018 appendix and aggregate 2019–2020 tables were treated as separate source components because equivalence of their record definitions, denominator construction, and available variables could not be demonstrated. Earlier Turkish datasets and publications from other countries were used only for contextual interpretation and surveillance mapping and were not pooled with the primary data.

The primary objective was to audit record definitions, denominators, missingness, category changes, and clinical-field linkage while describing the demographic and temporal distributions in the detailed 2014–2018 UZEM records. Secondary objectives were to report the non-equivalent 2019–2020 counts separately and to identify fields that could improve future scorpion-sting surveillance.

## 2. Results

### 2.1. Data Availability and Internal Consistency

The dedicated appendix reported 7055 scorpion-sting records during 2014–2018 and supplied the detailed variables used in the primary analysis. The general annual poison-group tables separately reported 1451 records in 2019 and 1729 in 2020, but provided no comparable scorpion-specific demographic, geographical, exposure, symptom, or management tables. The later counts were therefore retained only as a separate temporal extension.

The audit scope and 15 classified findings are summarised in Table 1. Supplementary Table S1 supplies finding-level provenance and decisions, and Supplementary Data S1 provides the corresponding machine-readable analytic and audit records.

The 15 findings included the following numerical conflicts. The 2018 dedicated appendix reported 1460 scorpion records, whereas a general annual table reported 1462. The report stated that 1465 scorpion records represented 0.63% of 213,533 poisoning records in 2017; direct calculation yields 0.69%. The 2019 report classified 140,280 calls by reason as cases, while several demographic and poison-group distributions totalled 217,936. The appendix conclusion also differed from detailed tables for the 2018 female and male percentages, physician-caller percentages in 2017–2018, and the 2018 percentage contacted within 60 min. The five absolute differences ranged from 0.03 to 0.07 percentage points; the largest was the 2017 physician-caller difference between 78.63% in the conclusion and 78.70% in Table 184 of the UZEM report [14]. Detailed-table values were retained. The complete register documents the source, decision, and inferential effect for each finding (Supplementary Table S1).

### 2.2. Annual Record and Demographic Distributions, 2014–2018

**Figure 2:**
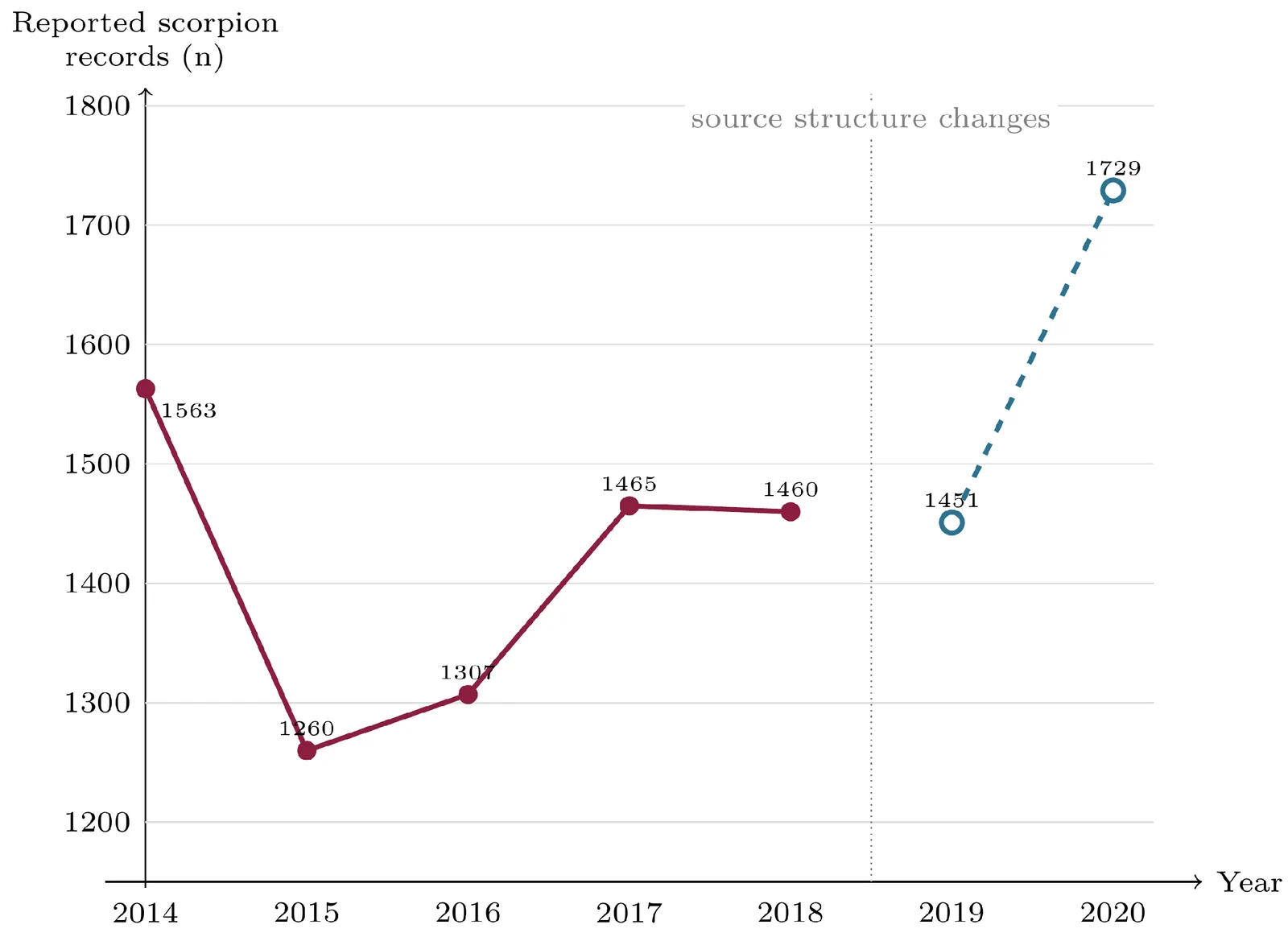
Scorpion-sting records reported by UZEM. Solid line: dedicated scorpion appendix (2014–2018). Dashed line and open markers: general annual poison-group tables (2019–2020). The two source components should not be treated as a single homogeneous time series.

Annual counts ranged from 1260 in 2015 to 1563 in 2014 (Table 2). The sequence decreased between 2014 and 2015, then increased to approximately 1460 records in 2017–2018. The separately sourced 2019 value was similar, whereas the 2020 general table showed 1729 records (Figure 2). Because the source component changed after 2018, this final increase was not subjected to trend testing.

**Figure 3:**
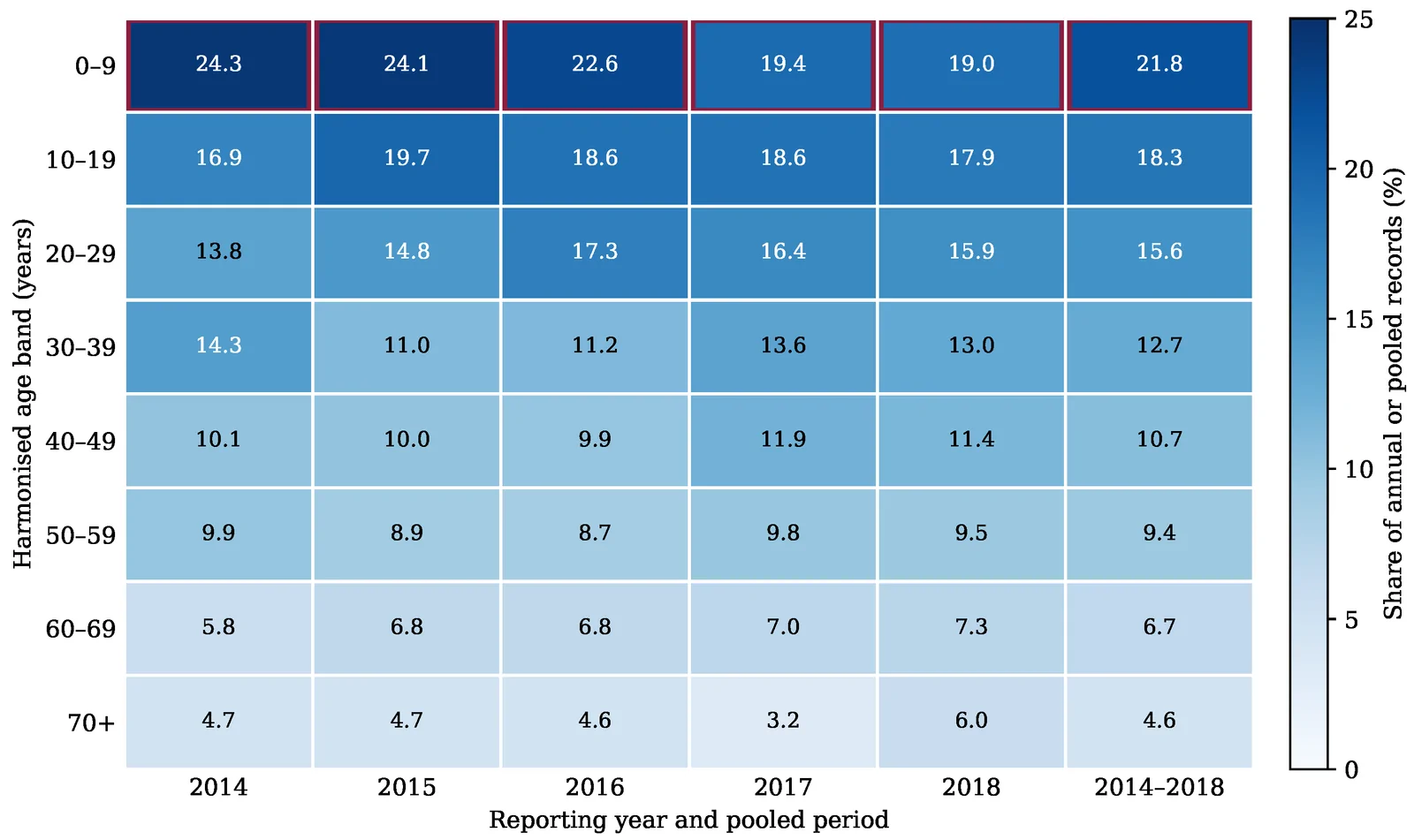
Harmonised age-band distribution of detailed UZEM scorpion-sting records, 2014–2018. Values are percentages of the full annual or pooled record total; outlined cells identify the largest band in each column. The 70-years-and-older category is open-ended. Complete counts and percentages, including unknown age, are reported in Supplementary Table S2. The figure was replotted from Supplementary Data S1 using Python 3.12.14/Matplotlib 3.11.2, with OpenAI Codex assistance in revising the plotting code; all displayed values are derived from the reported data.

Across 2014–2018, 3599 records (51.01%) involved males, 3443 (48.80%) involved females, and 13 (0.18%) had no reported sex. Male records predominated in 2014–2015, followed by a small female majority in 2016–2018. The published age tables used single-year categories for ages 0–4, five-year categories for ages 5–19, and 10-year categories from age 20 onward. Directly selecting the largest published cell therefore favoured the wider adult categories: 20–29 years was the largest original category in four years and 30–39 years in 2014. After harmonisation into comparable 10-year bands, however, 0–9 years was the largest band in every year, accounting for 1541 records (21.84%) overall (Table 2; Figure 3; Supplementary Table S2).

Table 3 quantifies published missing or non-specific entries using each year’s record total. Unknown age totalled 11 records, and unreported sex totalled 13; the 2015 sex value is a calculated residual. Exposure-setting labels differed in 2014–2016. In 2017–2018, no separate unknown category was published for setting or contact delay, so completeness cannot be inferred. Comparable fields were unavailable for 2019–2020.

Home, caller, and contact-delay fields showed abrupt changes between 2016 and 2017. Reporting categories also changed, but the physician category was present in both periods; the extent to which the observed shifts reflect coding, contact behaviour, or both could not be determined (Table 4).

### 2.3. Monthly Distribution of Reported Records

**Figure 4:**
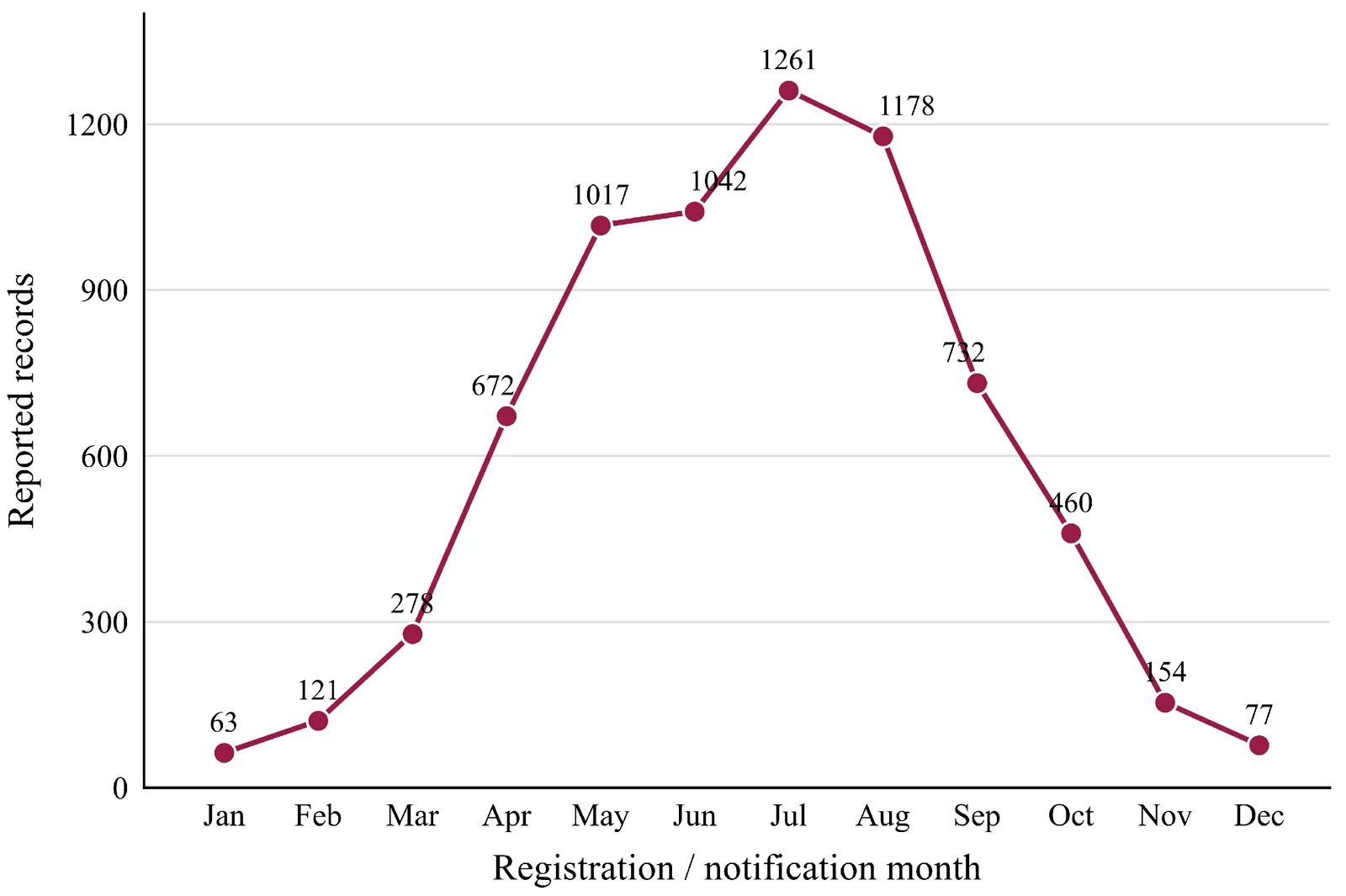
Combined monthly distribution of UZEM scorpion-sting records, 2014–2018.

Reported records peaked in July (1261; 17.87%), and May–September accounted for 5230 records (74.13%) of the five-year total (Figure 4). Because the source labels this field as registration or reporting month, the distribution represents reporting seasonality.

### 2.4. Scorpion Records Within Annual UZEM Activity

Within the totals reported in the selected annual source distribution tables, scorpion records represented less than 1% of the reported distribution-table total and approximately 41–59% of animal-exposure records (Table 5). These proportions describe the composition of UZEM’s recorded activity, not the proportion of all poisonings or animal exposures occurring in Turkey. They were not pooled because record definitions and denominators were not demonstrably stable across years.

### 2.5. Geographical Reporting Patterns

The province with the largest crude number of records was Ankara in 2014, Malatya in 2015, and Istanbul in 2016–2018. In 2017, Central Anatolia (272; 18.57%) and Eastern Anatolia (270; 18.43%) had similar shares; Eastern Anatolia ranked first in 2018 (257; 17.60%). These are crude poison-centre reporting counts.

### 2.6. Clinical-Field Completeness and Management Recommendations, 2017–2018

Comparable symptom and management tables were available only for 2017–2018. No sign or symptom was reported for 741 of 1465 records (50.58%) in 2017 and 549 of 1460 (37.60%) in 2018. Pain was the most frequently listed symptom (258 and 298 entries), while nausea or vomiting was listed in 33 and 52 records. Table 180 of the UZEM report [14] published year-specific top-ten lists plus “other”; a category absent from one year’s list was therefore coded as not published rather than zero. Systemic examples included tachycardia and hypertension in 2017 and hypotension and dizziness in 2018. Categories were non-exclusive, so values are reported as entries per 100 annual records. Patient-level symptom proportions were not inferred because a unique-event denominator and the completeness of symptom assessment could not be verified.

Symptomatic support was the largest published recommendation entry (1058 in 2017 and 926 in 2018). Other entries included tetanus prophylaxis, decontamination, electrocardiography/monitoring, referral, ward observation, intensive-care assessment, and antivenom (Table 6); all published list categories are supplied in Supplementary Data S1. Antivenom was recommended in 145 records in 2017 and 83 in 2018 (9.90 and 5.68 per 100 annual records), while post-administration feedback was reported separately for 71 and 46 records. Feedback was incomplete and unlinked to severity, dose, adverse reactions, or outcome. Accordingly, these tables describe clinical documentation and poison-centre advice, not antivenom effectiveness.

### 2.7. International Publication-Level Surveillance Mapping

Twelve candidate publications were assessed, six were excluded according to the eligibility criteria, and six publications were retained. The retained articles were heterogeneous examples of what particular analyses reported; they were not audits of the complete routine capacity of six national poison-control systems and were not used to compare disease frequency. Table 7 summarises the main design contrasts; the complete field-oriented matrix is Supplementary Table S3.

The United States analysis described 185,402 human scorpion exposures after excluding duplicate records, confirmed non-exposures, and records outside the specified geographical scope [15]. The French multicentre study screened 975 files and retained 624 patients stung by species native to French territories [16]. Morocco’s publication described several reporting streams within a national toxicovigilance programme, including a dedicated scorpion-information stream, rather than one uniform patient-level dataset [17].

The South African publication analysed 740 consultation entries, comprising 725 human and 15 animal exposures; repeat calls about the same exposure were merged, and incomplete forms were retained with the affected variable treated as unknown [18]. The Australian prospective study recruited 192 candidate patients but restricted its principal analysis to 95 definite human stings for which the scorpion was immediately collected and identified by an expert [19]. The Israeli publication retrospectively reviewed 225 human calls and reported clinical-severity groups and presentation delay [20]. These designs demonstrate potentially useful fields, but a field produced through expert identification, prospective follow-up, or restrictive eligibility cannot be assumed to be routinely and completely available throughout the corresponding country’s poison-centre records.

### 2.8. Audit-Derived Draft Surveillance Framework

The audit and publication-level mapping yielded the draft framework in Table 8. Candidate fields were provisionally grouped as core or extended according to interpretive value and anticipated collection, linkage, or verification requirements. Supplementary Table S4 provides illustrative operational definitions and coding notes. This author-derived synthesis has not undergone formal consensus, feasibility testing, or clinical validation.

## 3. Discussion

### 3.1. Principal Findings

Detailed UZEM records were concentrated in warmer months: three-quarters were reported between May and September. Because the source field denotes registration or reporting month, this pattern describes service activity rather than verified sting-event timing. The age audit produced a more consequential interpretive change. Although 20–29 years was the largest original published category in four of five years, those tables mixed one-year, five-year, and ten-year intervals. After aggregation into comparable 10-year bands, ages 0–9 formed the largest band in every year and accounted for 21.84% of all detailed-period records. The five-year sex distribution was nearly balanced.

The 2014–2018 appendix and the 2019–2020 general tables were not pooled because equivalence of their record definitions, denominator construction, and available scorpion-specific variables could not be demonstrated. The two-record 2018 conflict and the corrected 2017 percentage remain separately reported audit findings, but they were not the principal basis for separating the periods. The detailed 2014–2018 series was therefore analysed separately, with 2019–2020 retained as a qualified extension.

### 3.2. Contextualisation with Non-Equivalent Turkish Evidence

The seasonal pattern is consistent with the earlier UZEM series for 1995–2004, in which July predominated [7], and regional clinical studies also repeatedly describe clustering in warmer months [9,22,23]. The earlier publication identified ages 20–29 as the largest published category (219/930; 23.55%), but Table 1 of the earlier study by Cesaretli and Ozkan [7] separated ages ≤4 (131 records) and 5–9 (111 records). In a post hoc regrouping of those published counts, ages 0–9 total 242/930 (26.02%), exceeding the 20–29 count [7]. Thus, sensitivity of the descriptive ranking to category width is evident in both publications; this contextual calculation does not establish age-specific risk or change between periods.

The differences in magnitude are substantial and informative. UZEM recorded 7055 records in its detailed 2014–2018 appendix, whereas national healthcare-facility notifications numbered 24,261 in 2005 [12]. Emek and colleagues reported 30,769 scorpion stings during 2015–2020 from the 38 provinces whose submissions met study standards [13]. These values reflect different reporting routes and coverage rather than a common national trend. Emek and colleagues also identified missing or non-standard provincial entries and recommended a registration system containing descriptive, antivenom, and exposure-location fields, independently supporting standardised event-level data [13].

The geographical contrast reinforces this interpretation. UZEM’s highest crude counts shifted from Ankara to Malatya and then Istanbul, while facility-based national studies have emphasised the Mediterranean, Southeastern Anatolian, and Aegean regions [12,13]. Population size, referral networks, UZEM awareness, ease of telephone consultation, caller composition, and regional care-seeking pathways are plausible contributors. In endemic southern areas, established local hospital services may also reduce reliance on UZEM. These mechanisms were not measured, and the province associated with a report should not be read as a verified sting location.

### 3.3. International Publication-Level Mapping

The international mapping helps interpret UZEM by showing how ascertainment and record design shape published results. The selected articles differed not only in ecology and healthcare pathways but also in their analytic unit and eligibility rules. The US study excluded duplicates, confirmed non-exposures, and records outside its geographical scope; the French study retained 624 of 975 screened files; and the Australian study reduced 192 prospectively recruited candidates to 95 definite, specimen-confirmed stings [15,16,19]. The resulting distributions therefore describe different selected record sets and cannot be treated as directly comparable measures of national burden.

The South African article further illustrates why the publication must not be equated with the whole information system: its 740 entries included 725 human and 15 animal exposures, repeat calls for the same exposure were merged, and incomplete forms were retained with missing variables classified as unknown [18]. Morocco reported several programme streams rather than one uniform patient-level cohort, while the Israeli article was a retrospective call/chart series [17,20]. These features are analytically important because human/animal scope, deduplication, missingness, expert confirmation, and follow-up determine what a reported denominator and field mean.

The transferable evidence is therefore limited to record-design features demonstrated within each publication. A reported severity grade, outcome, species identification, or contact delay shows that the field was available for that analysis, whereas an unreported item cannot be assumed absent from the underlying service. The proposed UZEM fields draw on these examples without attributing countrywide capacity.

### 3.4. Clinical Interpretability and Antivenom-Data Limitations

Local symptoms such as pain, redness, and oedema were the most frequently listed findings in 2017–2018, consistent with Turkish clinical series and the wider literature [7,24]. The UZEM tables also contained selected systemic findings and recommendations for monitoring, referral, ward observation, or intensive-care assessment. However, high symptom non-reporting and the absence of a severity grade limited clinical characterisation. This is particularly important for children, who are at greater risk of severe systemic toxicity and for whom Turkish studies have identified clinical and laboratory severity markers [8,25,26,27]. A recent Şanlıurfa university-hospital series, for example, linked 1041 presentations to antivenom administration, hospitalisation, intensive-care use, and survival, although it remained a single-centre dataset [28].

From a clinical perspective, the central issue is not simply that fields are missing, but that missingness changes the apparent phenotype of the cohort. A record with no systemic feature documented is not equivalent to a record in which autonomic, cardiovascular, respiratory, and neurological findings were actively assessed and found absent. Similarly, the number of pain entries cannot be used as the denominator for a severity distribution when symptom assessment was incomplete and multiple responses were permitted. A consistently defined severity measure recorded at first contact and at the worst observed point could allow clinicians to distinguish uncomplicated local stings from evolving systemic envenomation and relate that trajectory to age, body weight, delay, disposition, and outcome. The present study did not select or validate a particular scale; scale choice, timing rules, inter-rater reliability, and clinical utility require expert assessment and prospective pilot testing.

Pre-hospital measures and subsequent analgesia are another example of clinically relevant information that aggregate poison-centre tables cannot link. A retrospective Muğla emergency-department study recorded hot application, cold application, or no pre-hospital intervention in 229 adults and reported lower analgesic requirements among those using heat; however, its retrospective design and lack of standardised pain assessment preclude a causal efficacy conclusion [21]. For surveillance purposes, the transferable lesson is to record first-aid measures, baseline and repeated pain scores, and analgesics actually administered as separate fields.

The antivenom data illustrate a second measurement problem. Recommendations declined from 145 of 1465 records (9.90%) in 2017 to 83 of 1460 (5.68%) in 2018, but recommendation, confirmed administration, severity, and outcome were not linked for the same exposure event. The difference could reflect case mix, clinician practice, availability, reporting, or policy; the source cannot distinguish among these mechanisms. It therefore has no supported interpretation as a change in antivenom use, envenomation severity, outcome, or policy. Recent antivenom-development work from Morocco and North Africa also underlines the regional and species-specific context of product evaluation [29]. Future surveillance should link these treatment stages and report their completeness.

### 3.5. Surveillance and Practice Implications

Poison-centre data describe demand for toxicological advice and identify periods in which clinical services may require greater preparedness. The May–September concentration supports seasonal planning for clinician education, antivenom distribution, referral pathways, and public prevention messages. Estimating population burden would require complementary coverage and denominator data.

The framework in Table 8 separates advice, verified administration, and outcome at the exposure-event level. These links are necessary to interpret treatment pathways; the provisional core/extended tiers do not establish implementation priority. Definitions, codes, responsibilities, feasibility, and clinical utility require multidisciplinary review and prospective pilot testing.

### 3.6. Strengths and Limitations

The principal strengths are the use of an official national source, transparent separation of non-equivalent source components, recalculation of key percentages, independent author verification, explicit assessment of missingness, and a machine-readable audit trail. Turkish evidence and selected international publications provided clinical and record-design context.

The source consisted of aggregated tables with changing category structures, incomplete clinical linkage, no coverage denominator, and no validation against clinical files. Age harmonisation enabled comparison through age 69, but the 70-year-and-older group remained open-ended. Clinical detail was limited to 2017–2018, and shifts in exposure setting, caller, or contact delay may partly reflect recoding.

Initial extraction was performed by one author and independently checked for critical values and decisions by the second, rather than duplicated for every published cell. The international component mapped six heterogeneous publications and may have missed non-English or poorly indexed reports; study-specific eligibility, follow-up, scope, and missing-data practices limit transferability.

## 4. Conclusions

Within the comparable 2014–2018 appendix tables, records were concentrated in warmer months, and ages 0–9 formed the largest harmonised 10-year band, replacing the interpretation suggested by the unequal original categories. Non-equivalent record definitions, denominators, and variable availability required the 2019–2020 counts to remain separate; small numerical discrepancies remained separately reported audit findings. Clinical tables quantified documentation gaps and recommendations but did not support severity or antivenom-effectiveness analyses. The draft framework prioritises fields needed to connect exposure, clinical trajectory, treatment, disposition, and outcome.

## 5. Materials and Methods

### 5.1. Study Design and Reporting Framework

This was a retrospective, descriptive secondary analysis and source-level data-quality audit of aggregated, routinely collected poison-centre data. Reporting was structured with reference to the STROBE statement and the RECORD extension for studies using routinely collected health data [30,31].

### 5.2. Research Questions, Terminology, and Analytical Framework

Four questions were specified for the analysis: (1) which scorpion-specific demographic, temporal, geographical, exposure, clinical, and management variables were available in the detailed 2014–2018 appendix; (2) whether the aggregate 2019–2020 scorpion counts were structurally comparable with that appendix; (3) which inconsistencies, category changes, denominator problems, and missing fields limited epidemiological or clinical inference; and (4) which record-design features were demonstrated in selected international poison-centre publications and could address the identified limitations.

The following terms were used consistently. A *record* is a source-reported unit that cannot be assumed to represent one unique person; an *analytic ledger record* is an extracted source value, reported total, or explicitly derived value used in this audit. A *call* is a documented contact with UZEM, and an *exposure event* is the underlying incident to which one or more contacts may relate. A *recommendation* is advice issued by UZEM; *administration* means that a treatment was reported as given; and *feedback* is a subsequent report to UZEM after administration. *Sting* is used for a reported scorpion sting, *exposure* for the broader poison-centre context or when clinical effects are uncertain, and *envenomation* only when venom-related clinical effects are described.

Methodological validity was assessed through internal consistency, reproducibility of calculations, and the fitness of each field for the stated inference. A descriptive conclusion was retained only when its numerator, denominator, category definition, and source tables were identifiable and arithmetically reproducible. Findings were classified as non-estimable when interpretation required an unsupported assumption about unique-person linkage, national coverage, stable coding, an undocumented symptom assessment, complete event-linked administration or outcome, or the population at risk.

### 5.3. Primary UZEM Source and Study Periods

The sole primary data source was the Turkish Ministry of Health publication *National Poison Control Centre (UZEM) Reports for 2014–2020* [14]. The publication was treated as two analytically distinct source components:

1. The *Scorpion Poisoning Report, 2014–2018* (PDF pages 604–680), which contains scorpion-specific tables and formed the primary dataset.
2. The general UZEM annual activity reports for 2019 and 2020, which contain aggregate scorpion counts within poison-group tables and were used only as a supplementary temporal extension.

The published data originated from contacts recorded by UZEM, Turkey’s national poison-information service. The available tables classified records by demographic characteristics, reporting month, the province or region associated with the report, exposure setting, caller category, time from exposure to contact, reported signs and symptoms, and management recommendations. Management recommendations and limited post-antivenom feedback were reported as separate fields. The publication did not document a unique-event identifier, repeat-contact linkage, deduplication procedures, the data-entry form, database validation, or linkage with hospital records. These features could not be reconstructed; analyses therefore followed the terminology and inferential rules defined above.

### 5.4. Eligibility and Variables

We included all aggregate records that UZEM classified as scorpion poisoning or scorpion sting. For the detailed 2014–2018 period, the extracted variables were year, month, sex, age group, province or region, exposure setting, caller category, time from exposure to contact, reported signs and symptoms, and recommended management. For 2019–2020, only annual scorpion counts and same-year poison-group denominators were considered sufficiently available for description.

The source age tables used unequal intervals: ages 0, 1, 2, 3, and 4 were listed separately; ages 5–9, 10–14, and 15–19 used five-year groups; ages 20–69 used 10-year groups; and age 70 or older was open-ended. To avoid comparing cells with different widths, ages 0–4 and 5–9 were summed as 0–9 years, and 10–14 and 15–19 were summed as 10–19 years. The original 10-year adult categories were retained, age 70 or older was reported separately, and unknown age remained an explicit category. Both the original-category issue and the harmonised distributions were treated as data-quality findings.

Province values were interpreted as locations associated with UZEM records. For coding-sensitive variables, exact matches such as home and physician caller were retained, unknown remained explicit, and residual categories were not merged without a source crosswalk. Symptom and management tables permitted multiple entries per record and were analysed as non-exclusive categories.

All scorpion-specific fields required for the prespecified manuscript outputs were eligible; no eligible output field was sampled out. For 2019–2020, records were retained only when both the scorpion count and the relevant within-year denominator could be identified. A variable was pooled across years only when its category definition and table structure supported the same calculation.

### 5.5. Contextual Turkish Evidence

Evidence from Turkey outside the 2014–2020 UZEM publication was used to establish national context but was not merged with the primary dataset. The contextual sources included the earlier national UZEM series for 1995–2004, Ministry of Health notifications for 2005, and provincial health-directorate data for 2015–2020, together with regional taxonomic and clinical studies. These datasets differ in reporting route, geographical coverage, inclusion criteria, observation period, and record unit; some periods also overlap. They were therefore used to compare broad seasonal, demographic, species, and clinical patterns only, not to calculate a combined national total or time trend.

### 5.6. Data Extraction and Consistency Audit

Initial extraction was performed by one author. For every value used in a manuscript table, figure, or stated result, the extraction record documented the source component, table number, PDF page, variable label, numerator, denominator, published percentage, recalculated percentage where applicable, and the decision applied. The final ledger contained 191 analytic records: 184 linked directly to cited source locations and seven annual scorpion-share calculations derived from those records. Checks were conducted at three levels: arithmetic agreement with row or column totals; agreement between narrative summaries and detailed tables; and cross-year agreement of variable definitions and category structure. A second-pass verification was performed against the source PDF. A second author independently reviewed the primary counts, denominators, discrepancy decisions, provenance matrix, and manuscript interpretations against the cited source locations. Both authors resolved discrepancies by consensus and approved the final verified dataset and manuscript. This verification reduced transcription and interpretation risk but was not a complete duplicate extraction of every published cell.

Audit findings were recorded separately from analytic values. Fifteen findings were classified as one conflict between published values, one arithmetic inconsistency, five small summary/rounding discrepancies, four definition or coding ambiguities, and four structural limitations in the publication. A transcription-error category was retained conceptually but assigned zero findings because the publication did not reveal whether any conflict resulted from transcription rather than calculation, rounding, coding, or source-version differences. Supplementary Table S1 provides the field, period, source table and page, finding class, published or source value, verified or comparator value, decision, and effect on inference. The current Supplementary Data S1 contains 206 data rows plus one header row: 191 rows labelled analytic and 15 rows labelled audit_finding in the record_class column. An accompanying scope note documents this composition and identifies the current file as the submission version. Five-year totals were calculated only when annual tables used the same variable structure. The following analysis rules were applied:

- For detailed 2014–2018 analyses, the dedicated scorpion appendix was prioritised over general annual tables.
- If a published percentage differed from the value obtained from its stated numerator and denominator, the recalculated value was reported, and the discrepancy retained in a table note.
- Unequally sized source age categories were not ranked directly for substantive interpretation; they were first harmonised into 10-year bands wherever the published cells permitted exact aggregation.
- The 2019–2020 counts were not added to the 2014–2018 detailed total because equivalence of record definitions, denominator construction, and variable availability could not be established; small numerical discrepancies were audited separately.
- No missing-value imputation was performed.

### 5.7. Statistical Analysis

Counts and percentages were used for descriptive analysis. Recalculated percentages were obtained as 100×n/N and rounded to two decimal places. Combined 2014–2018 monthly and sex distributions were calculated by summing structurally equivalent annual cells and using the detailed-period total of 7055 records as the denominator. Harmonised age-band counts were calculated by exact summation of published cells and are reported completely in Supplementary Table S2. Clinical and management counts for 2017–2018 were expressed as entries per 100 annual records; multiple responses allowed a record to contribute to several rows. Patient-level symptom proportions were not inferred because a unique-event denominator and the completeness of symptom assessment could not be verified. Annual scorpion shares were calculated within each year’s source-table totals but were not pooled across years. No hypothesis tests or regression models were used because the objective was a descriptive source audit and the aggregate record structure did not support population inference. Incidence and age-standardised rates were not calculated because population-at-risk denominators were unavailable.

The geographical and age-distribution figures were regenerated and verified for the final production version using Python version 3.12.14 (https://www.python.org/) and Matplotlib version 3.11.2. Codex (OpenAI) assisted with plotting code; the figures were rendered from documented source data, and all plotted values and labels were checked against those sources.

### 5.8. Contextual Literature and International Publication-Level Mapping

A targeted narrative search of PubMed, Google Scholar, and reference lists was conducted through 31 August 2026 using combinations of *scorpion sting*, *scorpion envenomation*, *Türkiye/Turkey*, *epidemiology*, *poison centre*, *toxicovigilance*, and *antivenom*. The journal-specific *Toxins* search page was additionally screened using *scorpion envenomation*. Relevant public-health, species, clinical-management, and antivenom articles were used for contextual interpretation but were not added to the six-publication mapping unless they met its primary-data eligibility criteria. National Turkish datasets were prioritised; regional and paediatric studies were used to contextualise reporting seasonality and clinical severity. For the contextual 1995–2004 age comparison, the published ≤4 and 5–9 counts were summed and divided by the published total of 930; this post hoc calculation was not incorporated into the primary UZEM audit ledger. Exact database-specific query strings, individual execution dates, result counts, Google Scholar screening depth, and a contemporaneous duplicate-management log were not retained; the search is therefore reported as targeted narrative mapping rather than a fully reproducible systematic search.

The international component was a structured publication-level mapping. PubMed and Google Scholar were searched with combinations of *scorpion/scorpionism*, *poison control centre/center*, *poison information centre/center*, *toxicovigilance*, *national*, *multicentre*, and *epidemiology*. Reference lists of eligible articles were also screened. Eligible publications had to report a scorpion-specific series or notification programme; derive data from a national, multicentre, or formally established poison-information service; and provide at least three mappable record-design or surveillance domains. Single-hospital series, case reports, laboratory studies, non-scorpion-specific annual reports, reviews without primary data, and duplicate or superseded analyses were excluded.

Supplementary Data S2 contains 17 source records plus one search-metadata row. Twelve potentially relevant publications entered the mapping assessment, while five additional sources were classified as contextual_only and retained in the search log outside the mapping sample; retention in Supplementary Data S2 does not indicate inclusion in the six-publication mapping. Of the 12 mapping candidates, six were excluded, and six publications were retained from the United States, France, Morocco, South Africa, Australia, and Israel [15,16,17,18,19,20]. Selection and exclusion decisions are provided in Supplementary Data S2, and the complete attribute matrix is Supplementary Table S3. The comparison extracted record unit and selection, human/animal scope, study-specific augmentation, repeat-contact and missing-data handling, coverage, season and setting, species identification, severity, caller and delay, management, outcome, and denominator information. A feature was recorded only as demonstrated in the cited publication. Because study designs and ascertainment differed, no pooled estimate or rank ordering was undertaken. The observed UZEM gaps were then translated into a draft framework and provisionally classified as core or extended according to interpretive value and anticipated resource requirements. This author-derived classification was not developed through formal consensus or pilot testing.

For bibliographic traceability, Supplementary Data S2 also documents the six excluded candidates [32,33,34,35,36,37] and two additional contextual-only publications [38,39]; these eight publications were not included in the six-publication mapping.

## Figures and Tables

**Table 1:** Quantified scope and outputs of the UZEM source audit.

Audit Component	Number	Interpretation
Analytic ledger records	191	Primary UZEM-source values used in manuscript outputs; 184 source-linked and 7 derived
Named analytic variables	39	Annual, demographic, monthly, geographical, contextual, clinical, and management fields
Distinct numbered tables in analytic records	41	Tables represented by the 191 analytic records
Distinct numbered tables in the complete audit ledger	42	Analytic records plus finding-level source comparisons; not every cell in each table was duplicated
Conflicting published values	1	The 2018 annual scorpion total differed between two source tables
Arithmetic inconsistencies	1	The published 2017 percentage did not equal its numerator divided by its denominator
Small summary/rounding discrepancies	5	Female, male, two annual physician-caller, and contact-delay values differed from detailed tables
Definition or coding ambiguities	4	Denominator, record-unit, month-variable, and cross-year category issues
Structural limitations in the publication	4	Unequal age bands, later-year field absence, symptom missingness, and absent event linkage
Confirmed transcription errors	0	None assigned because the publication did not establish the error mechanism

**Table 2:** Selected characteristics of the detailed UZEM scorpion-sting records, 2014–2018.

Year	Records	Male, %	Female, %	Leading 10-Year Age Band, n (%)	Province with Most Reported Records, n
2014	1563	56.62	43.19	0–9, 380 (24.31)	Ankara, 86
2015	1260	54.76	45.24	0–9, 304 (24.13)	Malatya, 66
2016	1307	48.97	50.88	0–9, 295 (22.57)	Istanbul, 86
2017	1465	47.65	52.08	0–9, 284 (19.39)	Istanbul, 108
2018	1460	46.99	52.74	0–9, 278 (19.04)	Istanbul, 105

Age categories were harmonised into common 10-year bands; complete definitions and distributions are given in Methods Section 5.4 and Supplementary Table S2. Percentages use the full annual record total, so the displayed male and female percentages may not sum to 100% because records with unreported sex are omitted from those columns. Province values identify the province associated with the report, not a verified sting location or population-standardised risk.

**Table 3:** Published missing or non-specific entries by field and year, n (%).

Year	Records, N	Unknown Age	Unreported Sex	Non-Specific Exposure Setting	Unknown Contact Delay
2014	1563	4 (0.26)	3 (0.19)	6 (0.38)	18 (1.15)
2015	1260	1 (0.08)	0 (0.00) ^a^	10 (0.79)	10 (0.79)
2016	1307	4 (0.31)	2 (0.15)	21 (1.61)	16 (1.22)
2017	1465	2 (0.14)	4 (0.27)	– ^b^	– ^b^
2018	1460	0 (0.00)	4 (0.27)	– ^b^	– ^b^
2019	1451	NP	NP	NP	NP
2020	1729	NP	NP	NP	NP

Percentages use the annual record total (N); later years are separate source components. Non-specific setting means “place not selected” (2014), “unknown” (2015), or “place not specified” (2016). ^a^ Calculated residual after subtracting published male and female counts; no unreported-sex category was published. Other numerical cells, including the 2018 age zero, were explicitly reported. ^b^ No separate unknown/missing category was published; the dash is not a zero. NP, comparable scorpion-specific field not published. Neither an omitted missing category nor an unavailable field establishes complete reporting.

**Table 4:** Selected variables affected by category changes and their cross-period interpretation.

Year	Home exposure setting, %	Physician caller, %	Poison-centre contact within 60 min, %
2014	99.62	97.70	44.66
2015	99.21	97.14	40.32
2016	98.01	92.20	41.01
2017	68.12	78.70	59.18
2018	75.82	77.05	60.55
Field	Source categories, 2014–2016	Source categories, 2017–2018	Analytical handling
Exposure setting	Home plus place not selected (2014), unknown (2015), or place not specified, workplace, and school (2016).	Home and other places; no separate unknown category.	Retain home; do not merge changing residual categories or infer completeness.
Caller	“Applicants” heading; physician explicitly listed.	“Callers” heading; physician retained.	Retain physician; do not attribute the change solely to recoding.
Contact delay	Elapsed-time categories with an unknown row.	Elapsed-time categories without an unknown row.	Retain the 0–60-min category; do not infer completeness.

Source labels are translated into English. Percentages use each year’s full scorpion-record total. Contact delay is the interval from exposure to first contact with Turkey’s National Poison Control Centre (UZEM), not treatment delay. “Other places” includes workplaces, military units, schools, and similar settings.

**Table 5:** Within-year scorpion-sting records relative to totals reported in UZEM source distribution tables.

Year	Reported Source-Table Total	Animal-Exposure Total	Scorpion Records	Scorpion/Source Total, %	Scorpion/Animal Total, %
2014	199,681	2734	1563	0.78	57.17
2015	201,608	2130	1260	0.62	59.15
2016	218,010	2528	1307	0.60	51.70
2017	213,533	3279	1465	0.69 ^*a*^	44.68
2018	217,323	3254	1460 ^*b*^	0.67	44.87
2019	217,936 ^c^	3569	1451	0.67	40.66
2020	187,528	4016	1729	0.92	43.05

The first denominator column reproduces the total used in each year’s source distribution tables. Percentages are 100× the scorpion-record numerator divided by the displayed within-year denominator; no pooled total is presented. ^*a*^ The report states 0.63%; 1465/213,533 yields 0.69%. ^*b*^ The dedicated appendix reports 1460, whereas a general annual table reports 1462. ^c^ The 2019 report separately lists 140,280 calls classified by reason; 217,936 is the total in the cited demographic and poison-group distribution tables.

**Table 6:** Selected clinical-documentation and poison-centre management entries, 2017–2018.

Variable	2017 (N=1465)		2018 (N=1460)	
	n	Per 100 Records	n	Per 100 Records
No sign/symptom reported	741	50.58	549	37.60
Pain listed	258	17.61	298	20.41
Hyperaemia/redness listed	138	9.42	139	9.52
Oedema listed	89	6.08	95	6.51
Nausea/vomiting listed	33	2.25	52	3.56
Tachycardia listed	20	1.37	—	—
Hypertension listed	15	1.02	—	—
Hypotension listed	—	—	12	0.82
Dizziness listed	—	—	10	0.68
Symptomatic support recommended	1058	72.22	926	63.42
Tetanus prophylaxis recommended	209	14.27	342	23.42
Decontamination recommended	197	13.45	285	19.52
ECG/monitoring recommended	313	21.37	178	12.19
Health-facility referral recommended	248	16.93	106	7.26
Higher-centre transfer recommended	160	10.92	108	7.40
Ward observation recommended	67	4.57	82	5.62
Intensive-care assessment recommended	12	0.82	12	0.82
Antivenom recommended	145	9.90	83	5.68
Post-administration feedback to UZEM	71	4.85	46	3.15

Tables 180 and 181 of the UZEM report [14] published separate annual top-ten lists plus “other”; all published entries are supplied in Supplementary Data S1. An em dash means that a category was not included in that year’s top-ten list, not that its count was zero. Multiple responses allowed the same record to contribute to more than one row. Recommendation and post-administration feedback were distinct source fields.

**Table 7:** Condensed publication-level comparison of scorpion-exposure record construction.

Setting and Publication	Record Set and Selection	Ascertainment or Record Handling	Fields Highlighted in the Publication
Turkey, 2014–2020	Published aggregate tables; detailed appendix for 2014–2018 and annual poison-group totals for 2019–2020	Secondary audit; repeat-contact linkage not reported; field structure changed across periods	Demography, reporting month, crude geography, setting, caller, delay, selected symptoms, recommendations, and limited antivenom feedback
United States [15]	185,402 human-exposure records after geographical and exposure exclusions	Identified duplicates excluded; secondary NPDS analysis	Exposure site and effects, management site, healthcare-facility and intensive-care use, and outcome
France [16]	624 native-territory species exposures retained from 975 screened files	Retrospective multicentre review with study-specific species attribution	Circumstances, species, symptoms, management, and standardised envenoming classes
Morocco [17]	Programme-level counts from several reporting streams	Multiple surveillance and monitoring streams rather than one uniform cohort	Notifications, prevention activities, treatment logistics, and programme indicators
South Africa [18]	740 consultation entries: 725 human and 15 animal exposures	Repeat calls merged; incomplete forms retained with affected fields coded unknown	Caller, province, delay, severity, antivenom recommendation, and call-rate denominator
Australia [19]	95 definite stings retained from 192 prospective candidates	Immediate specimen collection, expert identification, and clinical follow-up	Species, setting, anatomical site, symptom duration, treatment, and outcome
Israel [20]	Retrospective review of 225 poison-information-centre calls/charts	Study-specific chart abstraction; repeat-contact handling not specified	Severity groups, symptoms, electrocardiographic findings, management, and presentation delay

The comparison unit is the cited publication, not the complete poison-control system or country. The detailed attribute-by-setting matrix is Supplementary Table S3; screening decisions are in Supplementary Data S2.

**Table 8:** Draft data framework for future scorpion-sting toxicovigilance: provisional core and extended fields.

Provisional Tier	Domain	Candidate Fields and Recording Point	Published UZEM Limitation Addressed	Illustrative Publication-Level Support
Core	Event and contact identity	Exposure-event ID; contact ID/type; timestamp. Link every contact to the same event.	Record terminology was unstable; event uniqueness, linkage, and repeat-contact handling were unverified.	Duplicate exclusion in the US and same-exposure call merging in South Africa [15,18].
Core	Basic exposure and risk context	Sting date/time; province; setting; anatomical site; age; sex; weight; pregnancy; comorbidity; contact delay. Retain traceable corrections.	Registration month was not event month; age, location, delay, and unknown-category structures varied.	Exposure and patient descriptors in US and French publications; delay in South African and Israeli publications [15,16,18,20].
Core	Clinical assessment and trajectory	Timestamped local/systemic findings coded *present*, *absent*, *not assessed*, or *unknown*; candidate severity at first contact and worst observation. No scale is endorsed.	Non-reporting, uncertain linkage, and absent assessed/absent coding prevented severity estimation.	Severity groupings in French and Israeli publications [16,20].
Core	Advice, treatment, care trajectory, and disposition	Advice; prior and subsequent treatment; antivenom status/time; current and highest care; transfer; final disposition. Update at each stage.	Recommendation, administration feedback, severity, care trajectory, and disposition were not complete linked histories.	Management and outcomes in the US; antivenom recommendation and delay in South Africa [15,18].
Core	Outcome and data quality	Closure and follow-up status; final outcome; resolution, sequelae, or death; last follow-up source/date; field completeness and missingness reason.	Outcomes and follow-up were unlinked; omitted unknown categories could not establish completeness.	Outcomes in US and Australian publications; explicit unknown handling in South Africa [15,18,19].
Extended	Species and enhanced exposure evidence	Photograph/specimen; identifier and confidence; geocoded location; rural/urban and environmental context.	Species was not linked to clinical records; province counts did not establish sting location or species.	Expert identification in Australia and species evidence from France [16,19].
Extended	Detailed treatment, linkage, and denominators	First aid; pain scores; analgesics; antivenom product, dose, batch, indication, reaction; laboratory/cardiac data; links to care, discharge, mortality, and denominator sources.	Detailed treatment, reactions, resource use, outcomes, and population at risk were not event-linked.	Treatment/outcome follow-up in Australia and US; pre-hospital fields in a Turkish study [15,19,21].

The core/extended designation is provisional and does not establish feasibility or implementation priority. “Illustrative support” identifies only a field or design feature reported in the cited publication; it does not show that the field is routinely available, systematically collected, or complete in UZEM.

## Data Availability

The original contributions presented in this study are included in the article/Supplementary Material. Further inquiries can be directed to the corresponding author.

## Supplementary Materials

The following supporting information can be downloaded at https://www.mdpi.com/article/10.3390/toxins18100454/s1: Table S1, quantified audit scope and finding-level register (supplementary_table_s1.pdf); Data S1, current machine-readable analytic ledger and audit findings, with its scope and version note (supplementary_data_s1.csv; supplementary_data_s1_readme.txt); Data S2, international surveillance-mapping search and selection log (supplementary_data_s2.csv); Table S2, harmonised age-band distribution (supplementary_table_s2.pdf); Table S3, detailed international publication-level mapping (supplementary_table_s3.pdf); Table S4, illustrative data-dictionary entries and coding notes (supplementary_table_s4.pdf). Completed STROBE and RECORD checklists are supplied as STROBE_checklist.pdf and RECORD_checklist.pdf.

## Abbreviations

The following abbreviations are used in this manuscript:

RECORD	REporting of studies Conducted using Observational Routinely collected health Data
STROBE	Strengthening the Reporting of Observational Studies in Epidemiology
UZEM	National Poison Control Centre (Ulusal Zehir Danışma Merkezi)
