# Supplementary material for: Poison-Centre Scorpion-Sting Records in Turkey, 2014–2020: A Data-Quality Audit
Source: Toxins (Basel). 2026 Oct 19;18(10):454. doi: 10.3390/toxins18100454 (PMC13709895; doi:10.3390/toxins18100454)
Supplement: Supplementary file 1 [file toxins-18-00454-s001.zip › RECORD_checklist.pdf]

Completed RECORD Checklist

Manuscript: *Poison-Centre Scorpion-Sting Records in Turkey, 2014–2020: A Data-Quality Audit*

RECORD is applied here as a reporting aid for a source-level audit of published aggregate routinely collected data. The investigators did not access the underlying UZEM database or individual health records. Items that require person-level linkage or validation are therefore marked not applicable or unavailable with a reason. Selection of the UZEM source components and audit-ledger records is kept separate from selection of the 12 candidate publications used in the international mapping.

Item	RECORD reporting requirement (paraphrased)	Location in final manuscript
1.1	Name the routinely collected data type and, where possible, the database or source report in the title or abstract.	Title and Abstract (first page).
1.2	State the geographic region and observation period in the title or abstract.	Title and Abstract (first page).
1.3	State whether database linkage was performed.	Methods Section 5.2 (p. 15) and Section 5.3 (p. 15). No database or person-level linkage was performed.
6.1	Describe how the study population or record set was selected, including classifications or algorithms.	Primary UZEM source components: Methods Section 5.3 (p. 15). Aggregate-record eligibility and harmonisation: Section 5.4 (pp. 15–16). Publication-map eligibility: Section 5.8 (pp. 17–18) and Supplementary Data S2.
6.2	Cite or report validation of classification methods; state explicitly when validation is unavailable.	Extraction verification and reconciliation: Methods Section 5.6 (pp. 16–17). Permissible inference: Section 5.2 (p. 15). Lack of clinical-file validation: Discussion Section 3.6 (p. 14).
6.3	If databases were linked, describe and display linkage and linked-record counts.	Not applicable: no databases were linked and no individual records were accessed (Methods Section 5.2 (p. 15); Section 5.3 (p. 15)).
7.1	Supply a complete list of classifications or rules used to define variables.	Methods Section 5.4 (pp. 15–16); extraction and audit rules in Section 5.6 (pp. 16–17); calculation rules in Section 5.7 (p. 17); Supplementary Table S1, Supplementary Data S1, and Supplementary Table S2.
12.1	Describe the investigators' level of access to the underlying data.	Methods Section 5.3 (p. 15): access was limited to the publicly available aggregate report; no underlying UZEM database or health record was accessed.
12.2	Describe data cleaning, reconciliation, and handling of inconsistent values.	Methods Section 5.6 (pp. 16–17) and Section 5.7 (p. 17); Results Section 2.1 (pp. 3–4) and Table 1 (p. 4); Supplementary Table S1 and Supplementary Data S1.
12.3	State whether person-, institution-, or other-level linkage was used and how linkage quality was assessed.	Not applicable: no linkage was conducted, so linkage quality could not be assessed (Methods Section 5.2 (p. 15); Section 5.3 (p. 15)).
13.1	Describe how the final analysed record set was derived, including data-availability and quality filters.	Source-level derivation: Results Section 2.1 (pp. 3–4) and Table 1 (p. 4); Methods Sections 5.3–5.4 (pp. 15–16) and Section 5.6 (pp. 16–17). Table 2 (p. 5) is not a selection-flow table. Publication mapping: Results Section 2.7 (pp. 9–10), Methods Section 5.8 (pp. 17–18), and Supplementary Data S2; it did not derive the UZEM ledger.
19.1	Discuss implications of using data not created for the research question, including misclassification, missingness, and changing structure.	Discussion Section 3.6 (p. 14); clinical interpretability in Section 3.4 (p. 13); permissible inference in Methods Section 5.2 (p. 15).
22.1	Explain how readers can access supplemental data, protocols, or code.	Supplementary Materials statement (p. 18) and Data Availability Statement (p. 19). Supplementary Tables S1–S4 and Data S1–S2 are available with the published article.

Checklist updated on 19 October 2026 to match the published article. Section, table, figure, and page references follow the published version; supplementary items use the journal's numbering (Tables S1–S4 and Data S1–S2).
