# Supplementary material for: Poison-Centre Scorpion-Sting Records in Turkey, 2014–2020: A Data-Quality Audit
Source: Toxins (Basel). 2026 Oct 19;18(10):454. doi: 10.3390/toxins18100454 (PMC13709895; doi:10.3390/toxins18100454)
Supplement: Supplementary file 1 [file toxins-18-00454-s001.zip › STROBE_checklist.pdf]

Completed STROBE Checklist

Manuscript: *Poison-Centre Scorpion-Sting Records in Turkey, 2014–2020: A Data-Quality Audit*

This checklist uses STROBE as a reporting aid for a source-level audit of published aggregate poison-centre tables. The manuscript is not an individual-patient cohort, case-control study, or cross-sectional sample. Requirements that depend on participant-level recruitment, matching, follow-up, or clinical outcomes are therefore marked not applicable with the reason stated. The 12-publication candidate flow belongs only to the separate international publication mapping and is not presented as a flow of UZEM patients or records.

Item	Topic	Reporting requirement (paraphrased)	Location in final manuscript
1a	Title/abstract	Identify the observational design and routinely collected data source.	Title and Abstract (first page); source-level audit design: Methods Section 5.1 (pp. 14–15).
1b	Title/abstract	Give a balanced summary of methods and findings.	Abstract (first page).
2	Background	Explain the scientific background and rationale.	Introduction (pp. 1–3).
3	Objectives	State the primary and secondary objectives.	Introduction (p. 3); Methods Section 5.2 (p. 15).
4	Study design	Present the main design elements.	Methods Section 5.1 (pp. 14–15).
5	Setting	Describe the source, geography, reporting setting, and periods.	Methods Section 5.3 (p. 15); contextual sources: Section 5.5 (p. 16).
6a	Participants/records	State eligibility and selection.	UZEM source components and aggregate-record eligibility: Methods Sections 5.3–5.4 (pp. 15–16). Publication-mapping eligibility: Section 5.8 (pp. 17–18) and Supplementary Data S2; it is not a UZEM patient-selection flow.
6b	Matching	Report matching where relevant.	Not applicable: no matched groups or individual-level comparison groups were constructed (Methods Section 5.1 (pp. 14–15); Section 5.7 (p. 17)).
7	Variables	Define extracted variables and their interpretation.	Methods Section 5.4 (pp. 15–16); Figure 3 (p. 6); Supplementary Data S1 and Supplementary Table S2.
8	Data sources/measurement	Give source and extraction methods for each variable.	Methods Section 5.3 (p. 15) and Section 5.6 (pp. 16–17); Supplementary Table S1 and Supplementary Data S1.
9	Bias	Describe measures addressing selection, misclassification, missingness, and extraction error.	Methods Section 5.6 (pp. 16–17); analytical safeguards in Section 5.2 (p. 15); Discussion Section 3.6 (p. 14).
10	Study size	Explain how the analysed set was determined.	No patient-sample-size calculation was applicable. Source-defined periods and operational audit scope: Results Section 2.1 (pp. 3–4) and Table 1 (p. 4); Methods Section 5.4 (pp. 15–16).
11	Quantitative variables	Explain groupings and calculations.	Methods Section 5.4 (pp. 15–16) and Section 5.7 (p. 17); Results Tables 2–6 (pp. 5–9); Figure 3 (p. 6); Supplementary Data S1 and Supplementary Table S2.
12a	Statistical methods	Describe all statistical procedures.	Methods Section 5.7 (p. 17).
12b	Subgroups and interactions	Describe subgroup or interaction analyses.	Not applicable: no subgroup, interaction, regression, or hypothesis-testing analysis was conducted (Methods Section 5.7 (p. 17)).
12c	Missing data	Explain handling of missing or unreported fields.	Methods Section 5.4 (pp. 15–16) and Section 5.6 (pp. 16–17); Results Section 2.2 (pp. 5–7) and Table 3 (p. 6); Section 2.6 (pp. 8–9) and Table 6 (p. 9). No imputation was performed.
12d	Sampling strategy	Account for complex sampling where applicable.	Not applicable: the analysis used source-defined published aggregate tables, not a probability or complex survey sample (Methods Sections 5.3–5.4 (pp. 15–16)).
12e	Sensitivity analysis	Describe sensitivity analyses.	No model-based sensitivity analysis was applicable. Conflicting values and non-comparable periods were handled by explicit audit rules (Results Section 2.1 (pp. 3–4); Methods Section 5.6 (pp. 16–17)).

STROBE checklist continued

Item	Topic	Reporting requirement (paraphrased)	Location in final manuscript
13a	Record flow	Report numbers assessed and included.	No individual UZEM records were screened or recruited. The two UZEM source components and 191-record audit ledger are reported in Results Section 2.1 (pp. 3–4), Table 1 (p. 4), and Methods Sections 5.3–5.4 (pp. 15–16). The publication map assessed 12 candidates and retained six (Results Section 2.7 (pp. 9–10)); this is not a patient flow.
13b	Exclusions	Give exclusion reasons.	Not applicable to individual UZEM records because no person-level screening was possible. Publication-level exclusions: Methods Section 5.8 (pp. 17–18) and Supplementary Data S2.
13c	Flow diagram	Consider a flow diagram.	No patient-record flow diagram was applicable. No PRISMA-style diagram was used because the international component was a structured publication mapping rather than a systematic review; its decisions are logged in Supplementary Data S2 (Methods Section 5.8 (pp. 17–18)).
14a	Descriptive data	Report demographic, temporal, geographical, and clinical-field characteristics.	Results Sections 2.2–2.6 (pp. 5–9) and Tables 2–6 (pp. 5–9); Figure 3 (p. 6); Supplementary Table S2. Table 1 (p. 4) reports audit scope, not demographic characteristics.
14b	Missingness	Report numbers with missing or unreported data.	Non-clinical missingness: Results Section 2.2 (pp. 5–7) and Table 3 (p. 6); note to Table 2 (p. 5) and Supplementary Table S2. Clinical non-reporting: Section 2.6 (pp. 8–9) and Table 6 (p. 9).
14c	Follow-up time	Summarise follow-up duration where relevant.	Not applicable: the published aggregate source did not establish longitudinal follow-up (Methods Section 5.1 (pp. 14–15); Section 5.3 (p. 15)).
15	Outcome data	Report counts or summary measures for outcomes.	Not applicable as a STROBE patient-outcome item: no linked clinical outcome was available (Results Section 2.6 (pp. 8–9); Methods Section 5.2 (p. 15)). Tables 2–6 (pp. 5–9) report source-field counts. Results Table 8 (p. 11) and Supplementary Table S4 are design materials (Section 2.8 (p. 11)).
16a	Main results	Give unadjusted estimates and clarify denominators.	Descriptive audit results: Sections 2.1–2.7 (pp. 3–10) and Tables 1–7 (pp. 4–10); audit-derived draft framework: Section 2.8 (p. 11) and Table 8 (p. 11); denominator and calculation rules: Methods Section 5.7 (p. 17); Supplementary Data S1. No adjusted estimates were produced.
16b	Category boundaries	Report boundaries for grouped quantitative variables.	Methods Section 5.4 (pp. 15–16); Results Table 2 (p. 5); Figure 3 (p. 6); Supplementary Table S2.
16c	Absolute risk	Translate relative effects into absolute risk where relevant.	Not applicable: no relative-effect or population-risk model was estimated, and population-at-risk denominators were unavailable (Methods Section 5.7 (p. 17)).
17	Other analyses	Report subgroup, interaction, or sensitivity analyses.	Not applicable: none were conducted (Methods Section 5.7 (p. 17)).
18	Key results	Summarise results in relation to objectives.	Discussion Section 3.1 (p. 12).
19	Limitations	Discuss bias, imprecision, missingness, source changes, and likely effects.	Discussion Section 3.6 (p. 14).
20	Interpretation	Provide cautious interpretation in light of limitations and other evidence.	Discussion Sections 3.1–3.6 (pp. 12–14); Conclusions (p. 14).
21	Generalisability	Discuss external validity and transferability.	Discussion Section 3.2 (p. 12), Section 3.3 (pp. 12–13), and Section 3.6 (p. 14).
22	Funding	State funding and funder role.	Funding statement (p. 19).

Checklist updated on 19 October 2026 to match the published article. Section, table, figure, and page references follow the published version; supplementary items use the journal's numbering (Tables S1–S4 and Data S1–S2).
