# Supplementary material for: Poison-Centre Scorpion-Sting Records in Turkey, 2014–2020: A Data-Quality Audit
Source: Toxins (Basel). 2026 Oct 19;18(10):454. doi: 10.3390/toxins18100454 (PMC13709895; doi:10.3390/toxins18100454)
Supplement: Supplementary file 1 [file toxins-18-00454-s001.zip › supplementary_table_s1.pdf]

Supplementary Table S1. Audit scope, provenance, and finding register

The operational audit scope was the set of values from the primary UZEM 2014–2020 publication used in a manuscript table, figure, calculation, or stated result; it was not a census or duplicate extraction of every cell in that publication. Contextual literature calculations were not incorporated into this primary-source ledger. Supplementary Data S1 labels analytic and finding-level rows explicitly; Table S1a quantifies these components. It contains 191 analytic records covering 39 named variables: 184 records are linked directly to cited source locations and seven are derived annual scorpion-share calculations. The analytic records cite 41 distinct numbered UZEM tables; after the source comparisons required for the finding-level records are included, the complete audit ledger spans 42 numbered tables. No discrepancy was labelled a confirmed transcription error because the publication did not establish which original entry, if any, resulted from transcription. The accompanying Supplementary Data S1 scope note identifies the current submission version and distinguishes it from superseded working extractions organised under a different row scope.

Table S1a: Quantified scope of the extraction and audit ledger.

Component	Number	Interpretation
Analytic records in Supplementary Data S1	191	Values used in manuscript outputs; not all published table cells
Directly source-linked analytic records	184	Each has a table or report location and PDF page
Derived annual scorpion-share records	7	Calculated from source-linked numerators and denominators
Named analytic variables	39	Includes annual, demographic, monthly, geographical, contextual, clinical, and management variables
Distinct numbered tables in analytic records	41	Tables represented by the 191 analytic records
Distinct numbered tables in complete audit ledger	42	Analytic records plus finding-level source comparisons; not a claim that every cell in each table was duplicated
Classified audit findings	15	Detailed in Table S1b and machine-readable in Supplementary Data S1
Confirmed transcription errors	0	None assigned because error mechanism could not be established from the publication

Table S1b: Finding-level audit register.

ID	Field and period	Source table/page	Finding class	Published or source value	Verified or comparator value	Decision	Effect on permissible inference
A1	Annual scorpion total, 2018	Table 12, PDF p. 50; Table 174, PDF p. 656	Conflicting published values	General annual table: 1,462	Dedicated appendix: 1,460	Retain 1,460 for detailed-period analyses and disclose both values	Prevents treating all annual source components as one internally uniform series
A2	Scorpion share of all poisoning records, 2017	Appendix conclusion, PDF p. 680	Arithmetic inconsistency	1,465/213,533 reported as 0.63%	Direct calculation: 0.686%, rounded to 0.69%	Report recalculated value and preserve the published value in the audit trail	Corrects the descriptive percentage; does not establish population incidence
A3	Female percentage, 2018	Table 176, PDF p. 660; conclusion, PDF p. 680	Small summary/rounding discrepancy	Conclusion: 52.78%	Detailed table: 52.74%; absolute difference 0.04 percentage points	Retain detailed-table value	Negligible substantive effect, but demonstrates source-version dependence
A4	Male percentage, 2018	Table 176, PDF p. 660; conclusion, PDF p. 680	Small summary/rounding discrepancy	Conclusion: 46.95%	Detailed table: 46.99%; absolute difference 0.04 percentage points	Retain detailed-table value	Negligible substantive effect, but demonstrates source-version dependence
A5	Physician-caller percentage, 2017	Table 184, PDF p. 676; conclusion, PDF p. 680	Small summary/rounding discrepancy	Conclusion: 78.63%	Detailed table: 78.70%; absolute difference 0.07 percentage points	Retain detailed-table value	Largest of the small summary differences; caller percentage must be tied to the selected table
A6	Physician-caller percentage, 2018	Table 184, PDF p. 676; conclusion, PDF p. 680	Small summary/rounding discrepancy	Conclusion: 77.02%	Detailed table: 77.05%; absolute difference 0.03 percentage points	Retain detailed-table value	Caller percentage must be tied to the selected table
A7	Contact within 60 minutes, 2018	Table 182, PDF p. 672; conclusion, PDF p. 680	Small summary/rounding discrepancy	Conclusion: 60.52%	Detailed table: 60.55%; absolute difference 0.03 percentage points	Retain detailed-table value	Negligible substantive effect, but the source location must be specified
A8	Annual denominator, 2019	Annual report, PDF p. 89; Tables 43–44, PDF pp. 99 and 102	Definition ambiguity	140,280 calls classified by reason	217,936 records in demographic and poison-group distributions	Use 217,936 only for calculations based on those distribution tables; do not equate the denominators	Prevents construction of a single 2019 case denominator
A9	Record unit, 2014–2020	Annual reports and scorpion appendix	Definition ambiguity	Terms corresponding to case, application, call, notification, and contact are used	Unique-person linkage and repeat-contact handling are not reported	Use *record*, not *patient*; do not deduplicate or pool on an assumed case definition	Prevents patient-level incidence and homogeneous seven-year cohort claims
A10	Monthly variable, 2014–2018	Tables 145, 154, 163, and 179; PDF pp. 613, 628, 642, and 666	Definition ambiguity	Earlier tables label the field as registration/reporting month	Equivalence to month of sting cannot be verified from the published tables	Interpret as monthly distribution of reported records and poison-centre activity	Supports service-demand seasonality only; sting-event seasonality remains indirect

Continued on next page

Table S1b continued

ID	Field and period	Source table/page	Finding class	Published or source value	Verified or comparator value	Decision	Effect on permissible inference
A11	Exposure setting, caller, and contact delay, 2014–2018	Tables 148–150, 157–159, 166–168, 178, 182, and 184; PDF pp. 616–618, 631–633, 645–647, 664, 672, and 676	Cross-period category change	Exposure-setting residuals changed from place not selected (2014), to unknown (2015), to place not specified/workplace/school (2016); contact-delay tables included unknown rows	The 2017–2018 setting table used home/other without a separate unknown row; physician remained explicit under a revised caller heading; contact-delay tables omitted an unknown row	Retain exact matches descriptively; do not merge changing residual categories; do not infer complete reporting from omission of an unknown row	Category change is established, but whether observed shifts reflect coding, contact behaviour, or both cannot be determined
A12	Age categories, 2014–2018	Tables 147, 156, 165, and 177; PDF pp. 615, 630, 644, and 662	Structural category design	Ages 0–4 listed singly; ages 5–19 in five-year bands; adults mainly in 10-year bands	Exact aggregation to comparable 10-year bands makes ages 0–9 the largest band in every year	Use harmonised bands for comparison; retain 70+ as open-ended and unknown as explicit	Reverses the earlier young-adult interpretation; does not estimate age-specific risk without population denominators
A13	Scorpion-specific detail, 2019–2020	Tables 43–44, PDF pp. 99 and 102; Tables 68–69, PDF pp. 178 and 180	Structural data absence in the publication	Annual scorpion totals are published	Comparable scorpion-specific demographic, exposure, symptom, and management tables are not published	Report annual counts as a separate extension only	Prevents a homogeneous detailed 2014–2020 analysis
A14	Symptom documentation, 2017–2018	Table 180, PDF p. 668	Structural missingness and multiple response	No symptom reported for 741 and 549 records; separate annual top-ten lists plus “other” were published	Multiple symptoms may be listed; absence from one year’s top-ten list is not a zero count	Describe entries per 100 records; show a justified compact selection in the manuscript and all published list categories in Supplementary Data S1	Prevents patient-level prevalence, phenotype, and severity estimates
A15	Treatment and outcome linkage, 2017–2018	Table 181, PDF p. 670; appendix conclusion, PDF p. 680	Structural linkage absence	Annual top-ten recommendation lists plus “other” were published; antivenom recommendations: 145 and 83; post-administration feedback: 71 and 46	Recommendation and feedback are separate but not complete or event-linked to severity, dose, adverse reaction, or outcome	Supply all published recommendation-list categories in Supplementary Data S1 and interpret them as poison-centre documentation, not treatment rates	Prevents assessment of utilisation, safety, treatment response, or antivenom effectiveness
