# Supplementary material for: Poison-Centre Scorpion-Sting Records in Turkey, 2014–2020: A Data-Quality Audit
Source: Toxins (Basel). 2026 Oct 19;18(10):454. doi: 10.3390/toxins18100454 (PMC13709895; doi:10.3390/toxins18100454)
Supplement: Supplementary file 1 [file toxins-18-00454-s001.zip › supplementary_table_s2.pdf]

Table S2: Harmonised age-band distribution of detailed UZEM scorpion-sting records, 2014–2018

Age band	2014	2015	2016	2017	2018	2014–2018
0–9	380 (24.31)	304 (24.13)	295 (22.57)	284 (19.39)	278 (19.04)	1,541 (21.84)
10–19	264 (16.89)	248 (19.68)	243 (18.59)	272 (18.57)	261 (17.88)	1,288 (18.26)
20–29	215 (13.76)	186 (14.76)	226 (17.29)	240 (16.38)	232 (15.89)	1,099 (15.58)
30–39	224 (14.33)	139 (11.03)	147 (11.25)	199 (13.58)	190 (13.01)	899 (12.74)
40–49	158 (10.11)	126 (10.00)	129 (9.87)	175 (11.95)	167 (11.44)	755 (10.70)
50–59	155 (9.92)	112 (8.89)	114 (8.72)	143 (9.76)	139 (9.52)	663 (9.40)
60–69	90 (5.76)	85 (6.75)	89 (6.81)	103 (7.03)	106 (7.26)	473 (6.70)
70 or older	73 (4.67)	59 (4.68)	60 (4.59)	47 (3.21)	87 (5.96)	326 (4.62)
Unknown	4 (0.26)	1 (0.08)	4 (0.31)	2 (0.14)	0 (0.00)	11 (0.16)
Total	1,563 (100.00)	1,260 (100.00)	1,307 (100.00)	1,465 (100.00)	1,460 (100.00)	7,055 (100.00)

Values are n (%). The source listed ages 0, 1, 2, 3, and 4 separately; ages 5–9, 10–14, and 15–19 in five-year groups; ages 20–69 in 10-year groups; and age 70 or older as an open-ended group. For comparability, ages 0–4 and 5–9 were summed as 0–9 years, and ages 10–14 and 15–19 were summed as 10–19 years. Percentages use the full annual or pooled denominator, including records of unknown age. The 70-years-and-older category is not a 10-year interval. These distributions describe UZEM records and are not age-specific population rates.
