# Supplementary material for: Poison-Centre Scorpion-Sting Records in Turkey, 2014–2020: A Data-Quality Audit
Source: Toxins (Basel). 2026 Oct 19;18(10):454. doi: 10.3390/toxins18100454 (PMC13709895; doi:10.3390/toxins18100454)
Supplement: Supplementary file 1 [file toxins-18-00454-s001.zip › supplementary_table_s3.pdf]

Supplementary Table S3. Detailed publication-level mapping of scorpion-exposure record construction and ascertainment

Panel A. Turkey, United States, France, and Morocco

Attribute	Turkey, 2014–2020	United States, 2005–2015 (Kang et al.)	France, 2011–2020 (Vaucel et al.)	Morocco, 2000–2011 (Rhalem et al.)
Record unit and selection	Published aggregate cell or total; detailed 2014–2018 appendix followed by aggregate 2019–2020 tables	NPDS human-exposure record; duplicates, confirmed non-exposures, and records outside the specified geography excluded	Poison-centre file; 975 screened and 624 native-territory species exposures retained	Programme-level counts from toxicological inquiries, general declarations, and a dedicated scorpion stream
Human/animal scope	Scorpion-specific record unit not explicitly defined	Human only	Human only	Human poisonings
Study-specific augmentation	None; secondary audit of published tables	Secondary analysis of reported NPDS fields; no study-specific examination	Retrospective multicentre review with species attribution for the study	Multiple programme reporting and monitoring streams; not one uniform research cohort
Repeat-contact management	Unique-event linkage and repeat-contact handling not reported	Identified duplicates excluded	Not specified in the publication	Not specified across streams
Missing/unknown handling	Explicit unknown categories varied by field and year; some variables were not published	Unknown values and reliance on caller reports acknowledged	Completeness handling not fully specified for every mapped field	Not specified uniformly across streams
Fields demonstrated	Demography, reporting month, crude geography, exposure setting, caller, delay, selected symptoms, recommendations, and limited antivenom feedback	Exposure site, effects, management site, healthcare-facility and intensive-care use, and outcome	Circumstances, species attribution, symptoms, management, and standardised envenoming classes	Scorpion notifications, prevention activities, treatment logistics, and programme indicators

Panel B. South Africa, Australia, and Israel

Attribute	South Africa, 2005–2014 (Marks et al.)	Australia, 2000–2002 (Isbister et al.)	Israel, 12-month series (Bentur et al.)
Record unit and selection	One consultation entry per exposure; 740 entries retained	Prospective candidate enrolment; 192 candidates and 95 definite stings retained after specimen collection and expert identification	Retrospective poison-information-centre call/chart; 225 calls reviewed
Human/animal scope	725 human and 15 animal exposures	Human only	Human only
Study-specific augmentation	Retrospective abstraction from standard consultation forms	Prospective research assessment, expert species identification, and clinical follow-up	Retrospective chart abstraction; no prospective study-specific examination reported
Repeat-contact management	Repeat calls for the same exposure merged into one entry	Not specified in the publication	Not specified in the publication
Missing/unknown handling	Incomplete forms retained; affected variable recorded as unknown	Uncertain or unverified candidates excluded from the definite-sting analysis	Not specified for all mapped fields
Fields demonstrated	Caller, province, delay, severity, antivenom recommendation, and call-rate denominator	Species, setting, anatomical site, symptom duration, treatment, and outcome	Severity groups, symptom profile, electrocardiographic findings, management, and presentation delay

The comparison unit is the cited publication, not the entire poison-control system or country. "Not specified" means not documented, not absent. A reported field may reflect restrictive eligibility, expert review, retrospective abstraction, or prospective follow-up and therefore does not establish routine or complete system-wide availability. Full references are provided in the main manuscript; screening and exclusion decisions are documented in Supplementary Data S2.
