# Supplementary material for: Poison-Centre Scorpion-Sting Records in Turkey, 2014–2020: A Data-Quality Audit
Source: Toxins (Basel). 2026 Oct 19;18(10):454. doi: 10.3390/toxins18100454 (PMC13709895; doi:10.3390/toxins18100454)
Supplement: Supplementary file 1 [file toxins-18-00454-s001.zip › supplementary_table_s4.pdf]

Supplementary Table S4. Illustrative data-dictionary entries for selected candidate fields

Field	Operational definition	Illustrative permitted values or format	Recording time	Primary data source
`exposure_event_id`	Pseudonymous identifier assigned once to a reported sting event and used to link related contacts.	Locally unique alphanumeric string; required; immutable; excludes direct personal identifiers.	Generated at first UZEM contact.	UZEM information system.
`contact_type`	Relationship of the current contact to the exposure event.	`initial`; `follow_up`; `correction`; `closure`.	At every contact.	UZEM operator and system timestamp.
`sting_datetime`	Best available date and time of the reported sting, distinct from contact time.	ISO 8601 date/time plus certainty: `exact`; `estimated`; `date_only`; `unknown`.	First contact; later correction retained with an audit trail.	Caller, clinician, or verified clinical record.
`symptom_state`	Assessment state for each specified local or systemic clinical feature.	`present`; `absent`; `not_assessed`; `unknown`; optional onset time when present.	First assessment and worst observed assessment.	Clinician or caller, with assessor role recorded.
`antivenom_recommendation_status`	UZEM advice on whether antivenom should be given, recorded independently of subsequent treatment.	`recommended`; `not_recommended`; `deferred`; `not_addressed`; `unknown`.	At the initial advice and any revised recommendation.	UZEM clinical-advice record.
`antivenom_administration_status`	Verified information on whether antivenom was actually administered.	`administered`; `not_administered`; `unknown`.	At each treatment update and event closure.	Administering facility or verified medication record.
`antivenom_administration_datetime`	Date and time of the first confirmed antivenom dose; not applicable only when non-administration was confirmed.	ISO 8601 date/time plus certainty: `exact`; `estimated`; `unknown` when dose time or administration status is unknown; `not_applicable` only with `not_administered`.	When administration or non-administration is verified, or the timestamp is corrected.	Administering facility or verified medication-administration record.
`highest_care_level`	Highest verified level of care reached at any time during the exposure-event trajectory.	`no_facility_care`; `ambulatory_care`; `emergency_observation`; `inpatient_ward`; `intensive_care`; `unknown`.	Updated at each verified care-stage change and finalised at event closure.	Treating facility or verified follow-up report.
`final_disposition`	Verified destination at the end of the latest documented care encounter, recorded independently of peak care intensity, clinical outcome, and follow-up status.	`home`; `discharged_home`; `admitted`; `transferred`; `died`; `unknown`.	At encounter disposition and event closure or the latest verified clinical update.	Treating facility or verified clinical report.
`clinical_outcome`	Clinical state at the latest verified assessment, recorded independently of encounter disposition and the administrative follow-up process.	`resolved`; `improving`; `persistent_symptoms`; `sequelae`; `death`; `not_assessed`; `unknown`.	At each verified clinical follow-up and event closure; retain the assessment date and source.	Treating clinician, facility record, or verified follow-up report.
`follow_up_status`	Status of the poison-centre follow-up process, recorded independently of clinical disposition.	`not_planned`; `open`; `completed`; `lost_to_follow_up`; `unknown`.	At each follow-up attempt and administrative closure.	UZEM operator and information system.

These entries illustrate how broad domains can be converted into operational fields; they are not a complete national data dictionary or a validated code set. Recommendation status, verified administration status, and administration date/time are stored independently and must not be inferred from one another. For administration time, `unknown` denotes unresolved administration or timing information, whereas `not_applicable` requires confirmed non-administration. Highest care level, final disposition, clinical outcome, and follow-up status are separate fields: for example, `intensive_care` may coexist with `discharged_home`, and `discharged_home` may coexist with persistent symptoms and an `open` follow-up. A transfer value denotes an encounter-exit transition, not a level of care or clinical outcome. Implementation planning should determine recording burden, mandatory or optional status, data-entry responsibilities, governance, and software workflow. The permitted values and sources are illustrative and require local expert review and prospective pilot testing before implementation.
